# Characterizing wild bird contact and seropositivity to highly pathogenic avian influenza A (H5N1) virus in Alaskan residents

**DOI:** 10.1111/irv.12253

**Published:** 2014-05-14

**Authors:** Carrie Reed, Dana Bruden, Kathy K Byrd, Vic Veguilla, Michael Bruce, Debby Hurlburt, David Wang, Crystal Holiday, Kathy Hancock, Justin R Ortiz, Joe Klejka, Jacqueline M Katz, Timothy M Uyeki

**Affiliations:** a Epidemic Intelligence Service, Centers for Disease Control and PreventionAtlanta, GA, USA; b Influenza Division, Centers for Disease Control and PreventionAtlanta, GA, USA; c Arctic Investigations Program, Centers for Disease Control and PreventionAnchorage, AK, USA; d Departments of Medicine and Global Health, University of WashingtonSeattle, WA, USA; e Yukon Kuskokwim Health CorporationBethel, AK, USA

**Keywords:** Alaska, H5N1, influenza

## Abstract

**Background:**

Highly pathogenic avian influenza A (HPAI) H5N1 viruses have infected poultry and wild birds on three continents with more than 600 reported human cases (59% mortality) since 2003. Wild aquatic birds are the natural reservoir for avian influenza A viruses, and migratory birds have been documented with HPAI H5N1 virus infection. Since 2005, clade 2.2 HPAI H5N1 viruses have spread from Asia to many countries.

**Objectives:**

We conducted a cross-sectional seroepidemiological survey in Anchorage and western Alaska to identify possible behaviors associated with migratory bird exposure and measure seropositivity to HPAI H5N1.

**Methods:**

We enrolled rural subsistence bird hunters and their families, urban sport hunters, wildlife biologists, and a comparison group without bird contact. We interviewed participants regarding their exposures to wild birds and collected blood to perform serologic testing for antibodies against a clade 2.2 HPAI H5N1 virus strain.

**Results:**

Hunters and wildlife biologists reported exposures to wild migratory birds that may confer risk of infection with avian influenza A viruses, although none of the 916 participants had evidence of seropositivity to HPAI H5N1.

**Conclusions:**

We characterized wild bird contact among Alaskans and behaviors that may influence risk of infection with avian influenza A viruses. Such knowledge can inform surveillance and risk communication surrounding HPAI H5N1 and other influenza viruses in a population with exposure to wild birds at a crossroads of intercontinental migratory flyways.

## Introduction

Since December 2003, highly pathogenic avian influenza A (HPAI) H5N1 viruses have spread to poultry and wild birds on three continents, have adversely affected the poultry industry in many countries, and have caused more than 600 reported human illnesses with nearly a 60% case-fatality risk.^1^ Rare, limited person-to-person transmission of HPAI H5N1 virus has been reported,^2^ but there is no evidence of sustained human-to-human transmission. Rather, the primary risk factors for human infections with HPAI H5N1 viruses are touching or close proximity with diseased or dead poultry.^3^,^4^

Wild birds are the natural reservoir for avian influenza A viruses,^5^ and HPAI H5N1 viruses have been detected in wild birds in Asia since 2005–2006.^6^ Wild migratory birds have been implicated in contributing to the spread of avian influenza A viruses beyond Asia,^7^ including HPAI H5N1 virus since 2005,^8^ particularly clade 2.2 viruses.^9^ Furthermore, exposure to dead wild birds has been implicated as the source of HPAI H5N1 virus infection of humans in Azerbaijan.^10^ Studies conducted during the 1997 H5N1 outbreak in Hong Kong demonstrated that asymptomatic and mild HPAI H5N1 virus infections can occur among poultry workers and cullers;^3^ however, very limited data are available about the magnitude and risk of infection with avian influenza A viruses among persons in close contact with wild birds.

Major migratory bird flyways exist between Asia and Alaska and raise the possibility that Alaska could be an area at risk for the introduction and spread of HPAI H5N1 viruses among wild birds. For this reason, surveillance for HPAI H5N1 and other avian influenza viruses among wild birds was undertaken in multiple locations in Alaska from 2006 to 2010 among priority species known to migrate between Asia and Alaska, and non-priority species which co-mingle with priority species in Alaska and may be secondarily exposed.^11^ Among the more than 55 000 live and hunter-killed wild birds sampled during the 5 years of surveillance, detection of avian influenza A viruses ranged from 0·8% to 2·4% depending on the year, and no HPAI H5N1 viruses were detected.^11^ In addition, in a further analysis of viruses isolated from northern pintails, a priority duck species for HPAI H5N1 sampling throughout the Yukon–Kuskokwim Delta, western Alaska, has been identified as a region with a high proportion of viruses with gene segments of Eurasian origin.^12^

Sport and subsistence hunting and other activities which place humans in close proximity to wild birds are common in Alaska. The US Fish & Wildlife Service estimates that there were 8800 active waterfowl hunters in Alaska during the 2010 season with a total harvest of 106 200 ducks and geese.^13^ Alaska also supports a large number of seasonal and permanent wildlife biologists from state and federal agencies. Alaska sport and subsistence hunters and wildlife biologists may have different risks of human infection with avian influenza A viruses from those described in previous studies conducted in Asia, Africa, and Europe. The objective of this study was to characterize both behaviorally and immunologically a population that could be exposed to avian influenza viruses from wild birds. In this manuscript, we report the types and frequency of bird contact that may be potential risk factors for infection with avian influenza A viruses among Alaska residents and whether there was any evidence of seropositivity to HPAI H5N1 virus among Alaskans with and without direct contact with wild birds.

## Materials and methods

We conducted a cross-sectional interview and serologic survey, recruiting a convenience sample of participants from six rural Alaska villages, the city of Bethel, and the municipality of Anchorage in Alaska during 2007–2008. We collected data from participants via interview regarding their exposures to wild birds and performed serologic testing for antibodies against a clade 2.2 HPAI H5N1 virus strain. Clade 2.2 is the HPAI H5N1 virus clade that has spread to regions outside of Asia since 2005,^9^,^14^–^16^ with documented infection of wild birds as far east as Western Siberia.^17^

### Ethics statement

This study was approved by the regional Alaska Native Health Boards and the Institutional Review Boards of the Alaska Area Native Health Service and the Centers for Disease Control and Prevention (CDC). Participation in this study was voluntary, and potential participants were informed about the study and the consent process. All adult participants provided written informed consent prior to enrollment. Children aged 5–17 years provided informed assent in addition to the written consent of their legal guardian.

### Study population

We recruited and enrolled persons from four groups that had regular contact with wild birds: (i) rural subsistence bird hunters and (ii) their family members, (iii) urban sport hunters, and (iv) wildlife biologists. Enrollment of rural Alaska subsistence bird hunters and their families occurred in six villages in the Yukon–Kuskokwim Delta region of western Alaska. For this study, a subsistence hunter was defined as an individual who participated in the hunting of birds and lived in a rural area of Alaska. We enrolled rural subsistence hunters ≥5 years of age who had hunted ≥1 time in the previous 2 years prior to enrollment and for at least 2 years during their lifetime, and their family members aged ≥5 years. Individuals aged 5–17 years were included in this study because children have been disproportionately affected by HPAI H5N1 virus^18^ and because many Alaskan Native people begin hunting at a young age.

**Figure 1:**
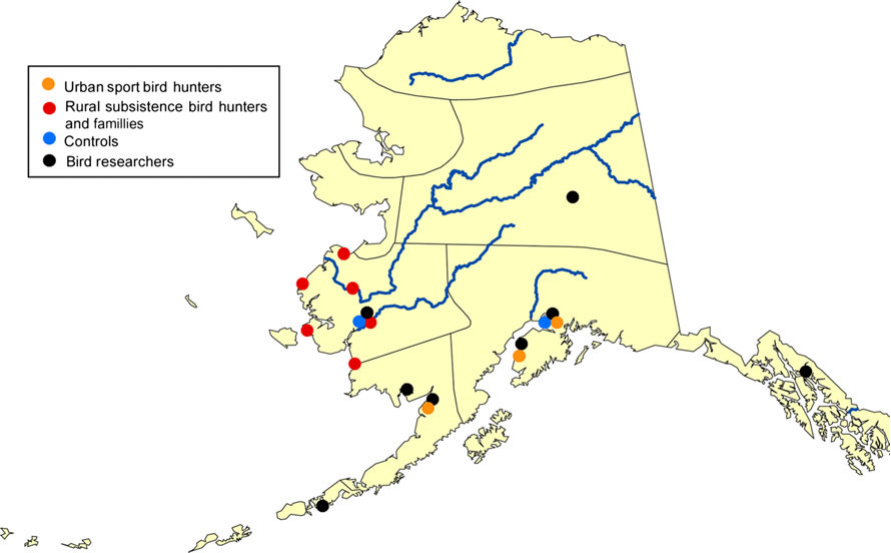
Approximate location of residence in Alaska of study participants according to their demographic group, 2007–2008.

We also enrolled sport hunters and wildlife biologists who resided in various cities and towns throughout Alaska (Figure1). We defined a sport hunter as a hunter who lived in an urban area and was recruited in Anchorage, the urban center of Alaska. To be eligible, sport hunters must have hunted for at least 2 years in their lifetime and must have hunted ≥1 time in the 2 years prior to enrollment. Wildlife biologists must have been engaged in fieldwork with wild birds for at least 1 field season in Alaska. A convenience sample of Alaskans who did not hunt or have contact with wild birds and resided in Anchorage or Bethel, the largest city in the rural Yukon–Kuskokwim Delta, was also enrolled as a comparison group. Anchorage and Bethel were selected as the site for recruitment because of the low likelihood of finding persons without bird contact in the six rural Alaska villages where subsistence hunters were recruited. Individuals who declined to submit a blood specimen were excluded from the survey because no serological testing could be performed.

### Data collection

Following informed consent, participants were given a standard verbally administered questionnaire and had a blood sample collected for serologic testing (5 ml from children aged 5–17 years and 10 ml from adults aged ≥18 years).

The questionnaire asked about localizing information (community, zip code, location of hunting), demographic information (age, gender, highest level of education, occupation), exposure to wild birds (at home and at work), type of contact with wild and domestic birds, cooking practices, and influenza vaccine history. We further asked about contact with specific species of waterfowl known to migrate between Alaska and Asia. The survey instrument is included as an appendix.

### Laboratory testing

Staff personnel obtained a blood specimen through venipuncture from participants using standard aseptic technique. The specimens were collected and labeled during survey administration; batched specimens were centrifuged to separate serum in the field for rural participants. Serum was refrigerated and transported to the CDC Arctic Investigations Program in Anchorage for storage in −30°C freezers. Sera were then shipped on dry ice to the CDC Influenza Division in Atlanta for serological testing of antibodies to HPAI H5N1 virus. Microneutralization (MN) and hemagglutination-inhibition (HI) assays were performed according to previously published procedures^19^ using A/Whooper swan/Mongolia/244/2005, a clade 2.2.1 HPAI H5N1 influenza virus, which was propagated in 10- to 11-day-old embryonated chicken eggs.

For the MN assay, sera were first heat-inactivated at 56°C for 30 minutes. Serial twofold dilutions of serum were tested in duplicates beginning with a 1:10 dilution. The titers were expressed as the reciprocal of the highest dilution that gave 50% neutralization. Sera yielding MN titers of ≥40 (equivalent to the WHO criteria of an MN titer of ≥80 using a starting dilution of 1:20) were tested further using two confirmatory assays, the HI, and Western blot, as recommended by the World Health Organization (WHO).^20^ MN titers of <10 were reported as 5 for the purpose of analysis.

For the HI assay, sera were first treated with receptor-destroying enzyme (RDE; Denka-Seiken, Tokyo, Japan), followed by heat inactivation at 56°C for 30 minutes. Serial twofold dilutions of serum were tested in duplicate beginning with a 1:10 dilution, and titers were expressed as the reciprocal of the highest dilution that gave complete inhibition of hemagglutination of 1% horse red blood cells by 4 hemagglutinating units of virus. Western blot analysis was performed as previously described using 4 ng/mm of a clade 2.1 recombinant HA protein based on A/Indonesia/05/2005 virus.^19^ A seropositive result was defined as an MN titer of ≥40 (equivalent to the WHO criteria of an MN titer ≥80) and a positive result by at least one confirmatory assay, including an HI titer ≥80 (equivalent to the WHO criteria of an HI titer ≥160) or a Western blot-positive result.^20^

### Data analysis

We calculated frequencies of persons seropositive for antibodies to HPAI H5N1 virus in the survey population by exposure group as well as frequencies of demographic and risk factors for the enrolled population. We compared the proportion of participants with a given characteristic using the Mantel–Haenszel chi-square test. All statistical analyses were performed in SAS version 9.2 (SAS Institute, Cary, NC, USA).

## Results

In six villages in Western Alaska, we enrolled 237 subsistence hunters and 229 of their family members from October 1 to November 7, 2007, all of whom were Alaska Native persons. For comparison, we also enrolled 164 urban sport hunters from January 11 to May 16, 2008, 82 wildlife biologists between January 10 to April 21, 2008 and 204 persons in Anchorage and Bethel with no bird exposure from October 24 to December 7, 2007. As shown in Table1, rural Alaskan subsistence hunters were predominantly male (91%) and ranged in age from 8 to 69 years (median: 23 years), while their other non-hunting family members were predominantly female (84%) and ranged in age from 5 to 85 years (median 20 years). Alaskan urban sport hunters were older than the subsistence hunters, with a median age of 47 years (range: 8–81), and similar in age on average to the wildlife biologists (median: 43·5 years). The predominant source of bird exposure was to wild birds, as very few persons in any exposure group reported poultry exposures, such as raising poultry at the house/yard or touching live poultry.

**Table 1:** Characteristics of study participants, by exposure group

Characteristic	Subsistence hunting		Other exposure groups		
	
	Hunters	Family members	Sport hunters	Wildlife biologists/researchers	No wild bird exposure
Number of participants	237	229	164	82	204
Age, median (range)	23 (8, 69)	20 (5, 85)	47 (8, 81)	43·5 (22, 63)	34·5 (6, 66)
Age group, *n* (%)					
5–19	91 (38)	110 (48)	11 (7)	0 (0)	32 (15)
20–39	92 (39)	61 (27)	44 (27)	37 (45)	84 (42)
40–59	46 (20)	46 (20)	86 (52)	40 (49)	71 (35)
60+	8 (3)	12 (5)	23 (14)	5 (6)	17 (8)
Gender, *n* (%)					
Male	216 (91)	37 (16)	153 (93)	54 (66)	60 (29)
Female	21 (9)	192 (84)	11 (7)	28 (34)	144 (71)
Household characteristics					
Has running water, *n* (%)	173 (73)	148 (65)	164 (100)	77 (94)	204 (100)
No. of people living in house, median (range)	6 (1, 17)	7 (2, 17)	3 (1, 9)	2 (1, 6)	3 (1, 12)
Level of exposure to wild birds, median (range)					
No contact	n/a	53 (23%)	n/a	n/a	204 (100%)
No. of years of contact*	13 (1, 60)**	10·5 (1, 75)***	31 (1, 71)**	15 (2, 40)†	n/a
Days per year with contact*	16 (1, 90)**	12 (1, 100)***	14 (1, 90)**	21 (0, 150)†	n/a
No. of birds handled per day*	5 (0, 100)**	4 (1, 20)***	4 (1, 30)**	20 (0, 500)†	n/a
Type of wild bird contact, *n* (%)*					
Hunting	237 (100)	0 (0)	164 (100)	33 (40)	n/a
Live capture of wild birds for research	0 (0)	0 (0)	0 (0)	82 (100)	
Prepared wild birds for eating	156 (66)	174 (76)	162 (99)	41 (50)	n/a
Slaughtered	112 (47)	127 (55)	160 (98)	28 (34)	n/a
Plucked or cleaned	156 (66)	173 (76)	159 (97)	41 (50)	n/a
Prepared or cooked	105 (44)	130 (57)	156 (95)	26 (32)	n/a
Collected wild bird eggs	148 (63)	79 (44)	3 (2)	8 (10)	n/a
Poultry exposures, *n* (%)					
Have live poultry at house/yard	0 (0)	0 (0)	13 (8)	3 (4)	3 (2)
Touched live poultry	9 (4)	10 (4)	33 (20)	17 (21)	12 (6)
Prepared raw poultry	16 (7)	22 (10)	18 (11)	8 (10)	4 (2)

* Among those who reported contact with wild birds.

** Length of exposure reported for their hunting activity only; excludes contact while preparing wild birds for consumption (plucking, butchering, cleaning, cooking).

*** Length of exposure reported for their preparation activity only (plucking, butchering, cleaning, cooking); this group had no hunting activity.

† Length of exposure reported for their professional contact with wild birds only; 33 wildlife biologists were also sport hunters which their time spent sport hunting was not included; medians with hunting activity included were 18 years, 30 days, and 20 birds.

Urban sport hunters had a longer reported median duration of exposure to wild birds (31 years) than either subsistence hunters (13 years) or wildlife biologists (15 years; *P* < 0·01). However, both subsistence and sport hunters reported similar median days per year of contact (16 versus 14, respectively) and median number of birds handled per day when hunting (5 versus 4, respectively). Wildlife biologists reported more days of wild bird contact per year (median, 21 days) and more birds handled per day (median, 20 birds). Many of the wildlife biologists also reported hunting wild birds (40%) in addition to their professional exposures (Table1).

Among the household members of rural Alaska subsistence hunters, 76% reported touching wild birds through handling and/or preparing birds for eating. These family members reported a median of 10·5 years of exposure to handling/preparing wild birds, preparing birds on a median of 12 days per year of a median 4 birds per day. Handling/preparing wild birds for consumption was also reported by subsistence (66%) and sport hunters (99%) and wildlife biologists (50%). Rural subsistence hunters and their family members also commonly reported collecting wild bird eggs, although this was rarely reported by sport hunters and wildlife researchers.

We also examined personal behaviors among participants while touching wild birds that could potentially affect their risk of infection with avian influenza A viruses. Rural subsistence and sport hunters frequently reported eating while hunting, although rural subsistence hunters were more likely to report smoking or chewing tobacco while hunting than sport hunters (*P* < 0·001; Table2). A small percentage of either rural subsistence or sport hunters reported wearing rubber gloves while hunting birds, although this was lower among subsistence versus sport hunters (6% versus 13%, *P* = 0·03). A similar percentage of subsistence and sport hunters reported washing hands during or after hunting (44% versus 38%, *P* = 0·28). Only one hunter reported wearing a face mask while hunting birds.

**Table 2:** Personal behaviors among persons exposed to wild birds by exposure group and exposure source

Exposure source and behavior	Subsistence hunting		Other exposure groups	
	
	Hunters	Family members	Sport hunters	Wildlife biologists/researchers
Hunting, *n* (%)				
Wear rubber gloves while hunting birds	15 (6)	n/a	21 (13)	4/31 (12)
Wash hands with soap or sanitizer while hunting birds	104 (44)	n/a	63 (38)	14/31 (42)
Eat while hunting	204 (86)	n/a	142 (87)	20/33 (61)
Smoke while hunting	92 (39)	n/a	24 (15)	3/33 (9)
Chew tobacco while hunting	87 (37)	n/a	18 (11)	5/33 (15)
Bird preparation, *n* (%)				
Wear gloves while cleaning/preparing birds	12 (8)	30 (17)	33/162 (20)	9/41 (22)
Wash hands with soap or sanitizer after cleaning/preparing birds	126 (81)	167 (96)	148/162 (91)	41/41 (100)
Professional duties, *n* (%)				
Wear gloves while handling live birds	n/a	n/a	n/a	51 (62)
Wash hands with soap or sanitizer after handling live birds	n/a	n/a	n/a	73 (89)

While cleaning or preparing birds for eating, most persons (>90%) reported washing hands after bird preparation activities, although it was less commonly reported among rural subsistence hunters (81%) than other exposure groups (*P* < 0·01; Table2). Rural subsistence hunters were also less likely to report wearing gloves while cleaning/preparing birds for eating (8%) than other groups (17–22%; *P* < 0·01). Only two participants reported wearing a face mask while cleaning and preparing dead birds. Wildlife biologists who hunted did not differ from other groups in their low reported use of gloves and hand washing while hunting birds, but during their professional duties, 62% reported wearing gloves while handling birds and 89% reported washing hands after handling birds.

No participants were seropositive based on WHO criteria for A/whooper swan/Mongolia/244/2005, an HPAI H5N1 clade 2.2.1 virus (0%, 95%CI: 0–0·4%). Geometric mean titers using the microneutralization assay ranged from 5 to 57. A small fraction of participants (2·2%, 95%CI: 1·4–3·4%) had low levels of detectable antibody with an MN titer ≥10, and this did not vary by study group. The one person with an MN titer >40 was determined not to be seropositive after confirmatory testing.

## Discussion

In our study, Alaska subsistence hunters and their families, sport hunters, and wildlife biologists reported a low frequency of exposure to poultry, but had substantial contact with wild migratory birds that may be infected with avian influenza A viruses. Fewer than half of participants reported personal protective behaviors such as wearing gloves or washing hands while touching wild birds, although wildlife biologists were more likely to report these behaviors during their occupational exposures to wild birds. This suggests that these populations are at risk of exposure to avian influenza A viruses infecting wild birds in the area, although none of the study participants had evidence of seropositivity to clade 2.2 HPAI H5N1 virus. This finding is consistent with the lack of detection of this virus in North America but also suggests no notable baseline cross-reactive seroprotection, potentially from exposure to other avian influenza viruses.

Epidemiological studies of human infection with avian influenza A viruses have been conducted among healthcare workers,^21^–^25^ social contacts,^26^–^28^^,^ and persons with poultry contact, such as poultry workers, bird cullers, and open bird market workers,^3^,^29^–^32^ generally during HPAI poultry outbreaks that resulted in human illness cases. Limited information is available, though, to assess exposures and risk factors for acquiring avian influenza A virus infections among persons with high levels of contact with wild birds, which are the natural reservoirs for avian influenza A viruses. Studies in waterfowl hunters, wildlife workers, and bird banders in the Midwestern United States that used a low antibody titer threshold to define a seropositive result (titer ≥10) have reported a low prevalence of seropositivity to low pathogenic avian influenza A viruses, more often associated with a long duration of wild bird exposure.^33^,^34^ The participants in our study reported contact with wild birds over a relatively short period of time each year for many years, although the levels of exposure varied widely between individuals.

Non-hunting family members of rural subsistence hunters across all ages also had substantial contact with wild birds, as a majority reported plucking, slaughtering, or otherwise preparing harvested birds. While we found no serologic evidence of infection with HPAI H5N1 virus in this group, plucking feathers from dead birds, which can facilitate aerosolization of virus, has been shown to be a possible source of HPAI H5N1 virus exposure from wild birds. For example, close contact with and de-feathering of infected wild swans were the most plausible exposures to HPAI H5N1 virus in a cluster of human HPAI H5N1 cases in Azerbaijan.^35^ In addition, in settings of household exposure to poultry, there is evidence that infections with HPAI H5N1 virus may have occurred through food preparation practices such as slaughtering, de-feathering, cleaning, and dressing poultry,^10^ all of which can result in virus aerosolization.

Studies of persons who have contact with either poultry or wild birds have shown variable levels of personal protective behaviors such as wearing gloves or eye protection or washing hands when handling birds, depending on whether the contact was occupational or recreational, or the nature of contact (hunting, farming, transporting birds, collecting specimens, etc.).^33^,^34^,^36^ In addition, frequent lack of running water in rural villages may contribute to decreased washing of hands by rural hunters. We found that among both subsistence and sport hunters, a minority of participants reported wearing gloves or washing hands while hunting and many reported eating or smoking while hunting wild birds, which could potentially increase the risk of infection when handling birds with avian influenza A viruses. When preparing birds for consumption, very few hunters or their family members also reported wearing gloves, although most did report washing hands following bird handling.

Wildlife biologists reported a higher number of days of contact with wild birds (median of 30 days when their hunting activity is included) and had contact with a greater number of birds per day than subsistence and sport hunters. Most of their job-related contact involved capture of live birds and may involve a different level of risk than in the hunting of wild birds. Additionally, the majority of wildlife biologists reported wearing gloves while handling wild birds during fieldwork, which may potentially mitigate their risk of infection if exposed to infected birds.

We used WHO criteria to define seropositive results of antibodies to HPAI H5N1 clade 2.2 virus.^20^ A few seroepidemiologic studies of antibodies to avian influenza A viruses, including HPAI H5N1 virus, have used a lower than WHO-recommended MN titer cutoff of ≥10 and no confirmatory testing as a measure of serologic response.^37^ However, defining a seropositive result using such a low antibody titer threshold, at the assay detection limit, may misclassify a previously non-infected person as previously infected. A small fraction of participants in this study (2·2%) had similar antibody titers in the absence of any known exposure to HPAI H5N1 viruses, which to date have not been found in North America.^38^ This suggests that these low titer serologic results may also reflect test variability near the limit of detection or detection of cross-reactive antibodies from exposure to other influenza viruses.

Our study is subject to a few limitations. First, we enrolled convenience samples of participants, and it is unclear whether those who participated may have been representative of the types and level of bird exposure of other community members in their respective exposure group. Second, neutralizing antibody responses are virus strain specific and may not detect an antibody response if the virus used in the serologic assays did not closely match circulating viruses that had infected participants. We chose to use a clade 2.2 HPAI H5N1 virus strain because it is the virus clade that has spread to regions outside of Asia since 2005, with documented infection of wild birds as far east as Western Siberia^17^ and because of the existence of migratory bird flyways from Asia to Alaska.^9^,^14^–^16^ Finally, studies have shown that antibody titers in asymptomatic seropositive individuals decline within 6–9 months and thus may not be detected unless infection occurred recently.^39^,^40^

Although HPAI H5N1 viruses have not been detected in Alaska or North America in wild birds, poultry, or people to date, this study provides information about the baseline HPAI H5N1 virus serologic profile among Alaska residents. In addition, we characterized many behaviors of Alaskans that result in direct contact with wild birds, potentially increasing their risk of infection when handling birds infected with avian influenza A viruses. Knowledge of these factors may help inform surveillance and risk communication surrounding the potential risk of infection with HPAI H5N1 viruses in a population with exposure to wild birds at a crossroads of intercontinental migratory flyways. In addition, as low pathogenic avian influenza (LPAI) A viruses have been identified among wild migratory birds in Alaska, additional serological analysis is ongoing in this survey to assess the risk of human infection with LPAI viruses, which may further our understanding of zoonotic transmission of avian influenza A viruses.

## Supporting Information

**Appendix1.** Questionnaire: Alaska Avian Influenza and Animal-Borne Disease Study.
