# Supplementary material for: Characterizing wild bird contact and seropositivity to highly pathogenic avian influenza A (H5N1) virus in Alaskan residents
Source: Influenza Other Respir Viruses. 2014 May 14;8(5):516–23. doi: 10.1111/irv.12253 (PMC4181814; doi:10.1111/irv.12253)
Supplement: Supplementary file 1 — Appendix1. Questionnaire: Alaska Avian Influenza and Animal-Borne Disease Study. [file irv0008-0516-SD1.docx]

**Appendix I: Questionnaire**

**Alaska Avian Influenza and Animal-Borne Disease Study**

**A.** **Personal Background Information**

**Date of Birth** ___/___/___ **Gender: □** M **□** F

**A1. Participant Group:**

**□** Rural Subsistence hunter [1]

**□** Rural Subsistence family [1.1]

**□** Sport hunter [2]

**□** Alaskan who does not hunt birds [3]

**□** Biologist/University researcher with wild bird contact [4]

**A3. What is your age in years?**______________

**A5. How much schooling have you completed?** ________________________________

**A6. Are you Alaska Native? □** Yes [1] **□** No [0]

**A7. How many people currently live in your house?** ________

**A8. How many rooms are there in your house (excluding bathrooms and closets)?** _____

**A9. What village/town/city do you live in?** ________________________

**A10. Do you have running water in your home? □** Yes [1] **□** No [0]

**B. Hunting exposures**

***Birds hunted/culled***

**B1. Over the past 2 years, have you hunted [*or had contact* with (touched, plucked, gutted, or cleaned)] any wild birds in Alaska?**

**□** Yes [1] If **hunter or wildlife biologist** go to B1a

If **plucker/cleaner** then skip to B12

**□** No [0] *Please skip to section C*

**□** Don’t know

**B1a. If YES, For how many years have you hunted [*or had contact* with] wild birds?**_____

**B1b. If YES, what kind?** Check all that apply

- Duck **□** YES [1] **□** NO [0]
- Northern Pintail **□** YES [1] **□** NO [0] **□** UNK
- Long Tailed Duck **□** YES [1] **□** NO [0] **□** UNK
- Green-winged Teal **□** YES [1] **□** NO [0] **□** UNK
- Mallard **□** YES [1] **□** NO [0] **□** UNK
- Sea duck **□** YES [1] **□** NO [0]
- King Eider **□** YES [1] **□** NO [0] **□** UNK
- Common Eider **□** YES [1] **□** NO [0] **□** UNK
- Dark geese **□** YES [1] **□** NO [0]
- Black Brant **□** YES [1] **□** NO [0] **□** UNK
- Emperor Goose **□** YES [1] **□** NO [0] **□** UNK
- White geese **□** YES [1] **□** NO [0]
- Snow Goose **□** YES [1] **□** NO [0] **□** UNK
- Brant tundra swans **□** YES [1] **□** NO [0]
- Common snipe **□** YES [1] **□** NO [0]
- Sandhill crane **□** YES [1] **□** NO [0]
- Shorebirds **□** YES [1] **□** NO [0]
- If YES, predominant species collected/contacted (if known):

________________________________________________

(shorebirds such as godwits, dunlins, plovers, sandpipers)

- Songbirds **□** YES [1] **□** NO [0]
- If YES, predominant species collected/contacted (if known):

________________________________________________

(songbirds such as warblers, wagtails, thrushes)

- Other _________________________________________________

***Extent of exposure***

**B2.** **In the last 2 years, on average, how many days do you hunt/[*or have contact* with] wild birds each year?** _________

**B3. In the last 2 years, on average, how many birds do you harvest [or collect] each day you are hunting [working with birds]?** ________________

***Location of exposure***

**B4. In which Alaska boroughs or census areas have you hunted/[or had contact with] wild birds in the last two years? [*Or where were the wild birds that you handled acquired?*]** *Check all that apply*

**□** Aleutians East Borough (Sand Point)

**□** Aleutians West Census Area

**□** Municipality of Anchorage

**□** Bethel Census Area

**□** Bristol Bay Borough (Naknek)

**□** Denali Borough (Healy)

**□** Dillingham Census Area

**□** Fairbanks North Star Borough (Fairbanks)

**□** Haines Borough (Haines)

**□** Juneau (City and Borough of)

**□** Kenai Peninsula Borough (Soldotna)

**□** Ketchikan Gateway Borough (Ketchikan)

**□** Kodiak Island Borough (Kodiak)

**□** Lake and Peninsula Borough (King Salmon)

**□** Matanuska-Susitna Borough (Palmer)

**□** Nome Census Area

**□** North Slope Borough (Barrow)

**□** Northwest Arctic Borough (Kotzebue)

**□** Prince of Wales-Outer Ketchikan Census Area

**□** Sitka (City and Borough of)

**□** Skagway-Hoonah-Angoon Census Area

**□** Southeast Fairbanks Census Area

**□** Valdez-Cordova Census Area

**□** Wade Hampton Census Area

**□** Wrangell-Petersburg Census Area

**□** Yakutat (City and Borough of)

**□** Yukon-Koyukuk Census Area

**For YK Delta Subsistence Hunters only**

**B4a. In which area of the YK Delta [list top 3] have you hunted/[or had contact with] wild birds in the last two years?**

________________________________________

________________________________________

________________________________________

***Hunting and wild bird exposures in the past 2 years:***

**B5. Do you wear a respiratory mask when handling wild birds while hunting [collecting]?**

**□** YES [1] **□** NO [0]

**B6. Do you wear rubber gloves when handling wild birds while hunting [collecting]?**

**□** YES [1] **□** NO [0]

**B8a. If YES, how often?** **□** Sometimes [1] **□** Always [2]

**B8b. If YES, what year did you start doing this?** ________

**B7. Do you ever wear gloves to keep your hands warm when handling wild birds while hunting [collecting]?**

**□** YES [1] **□** NO [0]

**B7a. If YES, how often?** **□** Sometimes [1] **□** Always [2]

**B8. Do you bring food with you while hunting [or when handling] wild birds?**

**□** YES [1] **□** NO [0]

**B9. Do you eat food while hunting [or when handling] wild birds?**

**□** YES [1] **□** NO [0]

**B10. D****o you ever wash your hands with soap or use hand sanitizer when you are hunting/ [or handling] wild birds?**

**□** YES [1] **□** NO [0]

**B12a. If YES, how often do you wash your hands after handling wild birds?**

**□** Sometimes [1] **□** Always [2]

**B11. While hunting [or handling] wild birds to you ever?**

**□** Smoke cigarettes **□** Chew tobacco

**B12. Within the last 2 years, did you do any of the following?**

B12a. Cut up [butcher] wild birds to be eaten? **□** YES [1] **□** NO [0]

B12b. Plucked feathers from wild birds? **□** YES [1] **□** NO [0]

B12c. Cleaned out organs of wild birds? **□** YES [1] **□** NO [0]

B12d. Prepared or cooked wild birds? **□** YES [1] **□** NO [0] If ALL = NO then go to B19

If ANY = YES, For how many years have you prepared [plucked, gutted, cleaned, or cooked] wild birds? _________

**B14. In the last 2 years, on average, how many days do you prepare [pluck, gut, clean, or cook] wild birds each year?** ______

**B15. In the last 2 years, on average, how many wild birds do you prepare [pluck, gut, clean, or cook] in a day when the hunter brings them to you?** ______

**B16. Do you wear rubber gloves when preparing (gutting/plucking etc.) wild birds to eat?**

**□** YES [1] **□** NO [0] (Skip to B17)

**B16a. If YES, how often? □** Sometimes [1] **□** Always [2]

**B16b. If YES, what year did you start doing this?** ________

**B17. Do you ever wash your hands with soap or use hand sanitizer after cleaning [i.e., plucking, gutting, removing organs of] wild birds?**

**□** YES [1] **□** NO [0]

**B17a. If YES, how often do you wash your hands after handling wild birds?**

**□** Sometimes [1] **□** Always [2]

**B18. Do you wear a respiratory mask when preparing wild birds to eat?**

**□** YES [1] **□** NO [0]

**B18a. If YES, how often? □** Sometimes [1] **□** Always [2]

**B19. Within the last 2 years have you gathered wild bird eggs?**

**□** YES [1] **□** NO [0]

**B20. Do you ever eat raw or undercooked [runny whites & yolk] eggs from wild birds?**

**□** YES [1] **□** NO [0]

**B21. Do you ever eat raw or undercooked [bloody] meat or blood from wild birds?**

**□** YES [1] **□** NO [0]

**B21a. If YES, what kind of birds?** ______________________

**B22. Of the wild birds that you harvest for your family, are [read through the options below] of them dressed and cleaned in your household [defined as primary residence, i.e., where they live]?**

**□** 100% All **□** 75% ¾ **□** 50% ½ **□** 25% ¼ **□** 0% none

**C. Poultry exposures and household characteristics**

**C1. Do you raise poultry (chickens, turkeys, quail) in your house or yard?**

**□** YES [1] **□** NO [0]

**C1a. If YES, how many poultry do you have in your house or yard?** ____________________

**C2. Within the last 2 years, have you touched live poultry (chickens, turkey, quail)?**

**□** YES [1] **□** NO [0]

**C3. Does your job involve working with live poultry?**

**□** YES [1] **□** NO [0]

**C3a. If YES, how many poultry do you have at your work?** ____________________

**C4. Within the last 2 years, did you do any of the following?**

C3a. Butchered poultry to be eaten? **□** YES [1] **□** NO [0]

C3b. Plucked feathers from poultry? **□** YES [1] **□** NO [0]

C3c. Cleaned out organs of poultry? **□** YES [1] **□** NO [0]

C3d. Prepared or cook raised poultry? **□** YES [1] **□** NO [0]

**C5. Do you ever eat raw or undercooked [bloody] meat or blood from poultry?**

**□** YES [1] **□** NO [0]

**C5a. If YES, what kind of birds?** ______________________

**C6. Do you collect rain water, ice or snow, to drink or cook with in your home?**

**□** YES [1] **□** NO [0]

**C6a. If YES, do you treat the water? □** YES [1] **□** NO [0]

**C6b. If YES, how is it treated?** ______________________ (ex. chlorine, boiling etc)

**C7. Do you own pets?**

**□** YES [1] **□** NO [0]

**C7a. If YES, please specify the type and number of animals: _________________**

**C7b. Do any of the pets live in the house (>50% of the its time) with you? □** YES [1] **□** NO [0]

**D. Vaccine History**

**D1. Did you have a flu shot or nasal spray this season?** **□** YES [1] **□** NO [0]

**D1a. If YES, when?** Please specify month and year _____/_________

**D2. Month and date if vaccinated in prior season:** _____/__________
